# Digital Storytelling to Reduce Hispanic Parents’ COVID-19 Vaccine Hesitancy: A Pilot Randomized Controlled Trial

**DOI:** 10.3390/vaccines13111093

**Published:** 2025-10-24

**Authors:** Sunny W. Kim, Fernanda Lozano, Michael Todd, Linda Larkey, Raheleh Bahrami, Kavya Juwadi, Alexis Koskan

**Affiliations:** 1 Edson College of Nursing and Health Innovation, Arizona State University, Phoenix, AZ 85004, USA; mike.todd@asu.edu (M.T.); Linda.Larkey@asu.edu (L.L.); rbahram3@asu.edu (R.B.); kjuwadi@asu.edu (K.J.); 2 College of Health Solutions, Arizona State University, Phoenix, AZ 85004, USA; flozano4@asu.edu (F.L.); Alexis.Koskan@asu.edu (A.K.)

**Keywords:** COVID-19, digital storytelling, vaccine hesitancy, community-based, Hispanic

## Abstract

Background/Objectives: Hispanic children in the U.S. experience disproportionately low COVID-19 vaccination rates, largely due to parental vaccine hesitancy. Digital storytelling offers a culturally relevant approach to address concerns through first-hand narratives. This study examined the feasibility and acceptability of a community-driven digital storytelling intervention to reduce vaccine hesitancy among Hispanic parents. Methods: Ten formerly vaccine-hesitant Hispanic parents developed digital stories about their reasons for vaccinating their child(ren) against COVID-19. We then enrolled 80 Hispanic parents whose children were not up to date with COVID-19 vaccines in a randomized feasibility trial. Intervention group participants (*n* = 40) viewed four digital stories selected by a community advisory board, while control group participants (*n* = 40) viewed four length- and format-matched videos about nutrition. Surveys were completed pre-intervention (T1), immediate post-intervention (T2), and at 2-month follow-up (T3). A subsample of intervention participants also joined focus groups at T3. Results: Qualitative data suggested that DST was an acceptable and engaging method of health education. Intervention group parents showed moderately larger increases in intention to vaccinate than did controls at T2 (*d* = 0.41) and T3 (*d* = 0.30). At T3, intervention group parents were more likely to have vaccinated their children than were controls (*OR* = 5.20, 95% CI = 1.63–16.57; *RR* = 3.10, 95% CI = 1.30–7.37). Conclusions: The community-driven digital storytelling intervention was feasible and acceptable, and findings suggest moderate effects on increasing vaccine intentions and uptake. Future work should evaluate its effectiveness in reducing parental vaccine hesitancy and, in turn, vaccine uptake for other childhood immunizations.

## 1. Introduction

As we continue to navigate the post-pandemic world, protecting children and adolescents against COVID-19 remains a global health priority. Although the severity of COVID-19 has decreased in many cases, the virus can still cause serious health issues, such as long COVID symptoms, hospitalizations, and, in rare cases, death, especially for children with underlying conditions such as asthma, obesity, or diabetes [1,2,3]. While the overall COVID-19 infection rates for children have decreased, Hispanic children still experience disproportionately higher hospitalization rates compared to their non-Hispanic White counterparts [4,5]. Specifically, Hispanic children under 12 years old are hospitalized at a rate 1.8 times higher than non-Hispanic White children in the same age group, and Hispanic adolescents are 1.5 times more likely to require hospitalization [4,5].

Vaccination remains a cornerstone for protecting populations against COVID-19. These vaccines demonstrate high efficacy in preventing infection and minimizing the severity of the disease. However, since June 2022, when the U.S. Food and Drug Administration (FDA) authorized COVID-19 vaccines for children under five, progress in achieving equitable vaccination rates has been uneven [6]. The most recent data from Kaiser Family Foundation’s COVID-19 Vaccine Monitor shows that approximately 21% of children aged 6 months–4 years, 39% of children ages 5–11, and 50% of those aged 12–17 have received at least one COVID-19 vaccine dose [6]. However, despite ongoing public health initiatives, vaccination rates for COVID-19 and influenza among children in underserved Hispanic communities remain relatively lower than non-Hispanic White counterparts. For example, data from the CDC [7] indicate that during 2023–2024, COVID-19 vaccination coverage for Hispanic children was slightly higher than among Black children but still lagged behind rates for non-Hispanic White and other non-Hispanic groups [7]. Vaccination rates are particularly low in border states such as Arizona (15.8%), where a significant proportion of residents are of Hispanic heritage [8].

This persistent disparity is primarily driven by vaccine hesitancy among Hispanic parents, rooted in a complex interplay of factors [9]. Research has identified several key drivers to Hispanic parents’ COVID-19 vaccine hesitancy, including misinformation, limited access to reliable Spanish-language information, cultural beliefs, and historical mistrust of the healthcare system [10,11]. Research with low-income Hispanic parents has identified multiple factors of COVID-19 vaccine hesitancy. Parents often question whether the vaccine was adequately tested for children, express fears about potential short- and long-term health effects (e.g., infertility, neurological complications, or disruption of immune system development), and voice concerns about vaccinating children at younger ages [12,13]. Additional barriers include perceptions that vaccination is unnecessary and broader mistrust of institutions such as government agencies, pharmaceutical companies, and the scientific community [14,15]. Traditional public health campaigns that rely on factual information and expert recommendations have had limited success in overcoming vaccine hesitancy within the Hispanic community [16,17]. Culturally tailored interventions that address specific concerns and leverage trusted community messengers are needed to increase vaccine confidence and uptake [18]. Digital storytelling presents a promising approach for delivering such culturally relevant messages, thereby promoting behavior change [19,20].

One emerging strategy is digital storytelling, which provides a culturally grounded and participatory means of health communication. Digital storytelling is a form of community-based participatory research in which individuals create short, first-person audiovisual narratives that combine spoken word, images, music, and text to convey personal experiences [21]. This blending of oral tradition and digital media has been widely used in education, community engagement, and health promotion to emphasize authentic voices and deliver messages that resonate within cultural contexts [19,22]. A growing body of work has examined how storytelling-based approaches may support vaccine uptake [23,24,25]. For example, a recent study by Maragh-Bass et al. [23] found that digital storytelling empowered young Black adults to make more informed COVID-19 vaccination decisions, with participants reporting high levels of acceptability for this intervention format. Beyond vaccination, individuals engaged in DST workshops frequently describe feelings of empowerment and personal value derived from sharing their narratives with their communities [26,27], which has also been observed in the context of COVID-19 vaccine decision-making among young Black adults [23]. Evidence from broader health research indicates that digital stories can stimulate positive health behavior change [24,25]. Building on this, scholars have suggested that the method holds strong potential for use in Hispanic communities [27,28,29]. Despite this promise, the role of DST in shaping Hispanic parents’ decisions about vaccinating their children against COVID-19 remains underexplored, highlighting a critical gap in the literature.

Our digital storytelling intervention is grounded in the Theory of Planned Behavior [30] and a culturally centered health promotion model, known as Narrative as Culture-Centric Health Promotion [20]. By integrating these frameworks, we propose that culturally resonant digital stories, featuring relatable examples of desired health behaviors, can promote emotional engagement (transportation into the story) and foster identification with both the characters and the narrative. These emotional and relational responses are expected to influence core Theory of Planned Behavior constructs, including attitudes toward vaccination, perceptions of social norms, and perceived behavioral control, which, together, can shape vaccine intentions and, in turn, vaccine uptake behavior. When applied to COVID-19 vaccination, the Theory of Planned Behavior suggests that individuals who hold positive attitudes about the vaccine, believe their social circle supports vaccination, and feel confident in their ability to vaccinate their children, are more likely to vaccinate their children against COVID-19.

Given the requirement of parental consent [31] and the critical role parents play in health-related decision-making [32], parents’ attitudes and intentions to vaccinate their children are key to promoting COVID-19 vaccination. Thus, the study aims were to (1) co-develop a digital storytelling intervention with Hispanic parents or caregivers of children who have already been vaccinated, (2) determine the feasibility and acceptability of this digital storytelling intervention, and (3) explore patterns of change in the Theory of Planned Behavior constructs, vaccine hesitancy, and vaccination behavior in the intervention group relative to those in a control group, among Hispanic parents of children who are not up to date with CDC recommended COVID-19 vaccine doses.

## 2. Materials and Methods

### 2.1. Design, Sample, and Recruitment

The study comprised two phases. In *Phase I*, we co-developed digital stories with formerly vaccine-hesitant Hispanic parents. The stories focused on the transformation of Hispanic parents from vaccine-hesitant to vaccine-accepting. Ten Hispanic parents were invited to spend two consecutive days (six hours each day) at an in-person workshop with our team to develop their narratives into digital stories. Participants were considered eligible for the study if they met all of the following conditions. First, they self-identified as Hispanic/Latino. Second, they were the biological parent or legal guardian (e.g., grandmother or other custodial caregiver) of at least one child under the age of 18. Third, participants reported that they were initially hesitant or reluctant to vaccinate their child(ren) against COVID-19 but ultimately chose to have them vaccinated. Finally, participants needed to be willing and able to engage in the digital storytelling intervention and complete study-related assessments. Importantly, English language fluency was not required. To maximize inclusivity and minimize barriers to participation, recruitment and intervention activities were facilitated by bilingual and bicultural community health workers who could communicate with participants in either English or Spanish. This approach allowed us to authentically engage a diverse pool of Hispanic parents and caregivers.

In *Phase II*, we employed a two-arm randomized controlled trial design to assess the feasibility and acceptability of the DST intervention compared to an information-only control group among Hispanic parents, examining their attitudes toward COVID-19 vaccination and intentions to have their child vaccinated (Clinicaltrials.gov ID: NCT06036134). Parents were recruited if they (a) self-identified as Hispanic/Latino, (b) were 18 years old or older, (c) were biological parents or legal guardians, and (d) had one or more vaccine eligible children aged 6 months to 17 years old who are not up-to-date with CDC-recommended COVID-19 vaccine doses. If an eligible parent had multiple children who had not received the COVID-19 vaccination, they were instructed to respond for the child with the most recent birthday. The study sample consisted of 80 Hispanic parents or legal guardians of children under 18 years old.

To recruit our target population, we partnered with two bilingual and bicultural (English/Spanish) community health workers from a local community health worker agency who work primarily with Spanish-speaking Hispanic adults, a strategy proven to be effective in our region. They recruited participants from December 2023 to October 2024. The study was approved by the Institutional Review Board (IRB# STUDY00017735).

### 2.2. Setting and Procedures

#### 2.2.1. DST Workshop (Phase I)

The ten formerly vaccine-hesitant Hispanic parents participated in one of two workshops (January and February 2024) to co-develop their stories. A certified digital storytelling workshop facilitator (lead investigator), bilingual community health workers, and study team members facilitated the in-person digital storytelling workshops. We created discussion guides and prompts to help participants think through their experiences transforming from COVID-19 vaccine-hesitant to vaccine-accepting [33]. Next, we facilitated a story circle where participants shared their stories of transformation with the group and received feedback from other participants and facilitators. Participants finalized their narratives at the end of the first day of the workshop. All activities were digitally recorded and transcribed.

On the second day of the workshop, the research team collaborated with workshop participants to select meaningful photos and images that effectively illustrated their narratives, choose music to accompany their stories, and create storyboards that combined the text of their stories, photos/images, and copyright-free background music. The team worked with participants to record their voiceovers, choose a title, create dedication statements, and add the images and background music to finalize their stories. Our team provided coaching, technical support, and assistance during the entire process to maximize their learning about digital editing, reduce their frustration, and ensure the stories are their personal and authentic expressions. The participants signed a story release form, permitting the sharing of their stories in public settings. Each participant received $300 USD for their time and effort in creating their stories, and they received childcare vouchers to participate in the in-person workshop.

#### 2.2.2. Community Advisory Board Input

Our team engaged a community advisory board comprising individuals from varied backgrounds, including local Latino community leaders, representatives from advocacy groups, healthcare professionals, specialists in maternal and child health, Hispanic parents whose children had been vaccinated against COVID-19, a nonprofit organization dedicated to immunization, a maternal and child health research team, the local and statewide department of health service, and a physician from a local children’s hospital [33]. Before the workshops, the community advisory board identified common reasons that Hispanic families were hesitant to vaccinate their children against COVID-19. After the digital stories were created, the community advisory board reviewed all stories and selected the four most compelling stories to use as intervention materials in the second phase of the study.

#### 2.2.3. Intervention and Survey Data Collection (Phase II)

Our community health workers assessed potential participants for eligibility using an eligibility screener. To ensure balance across study conditions, we used stratified randomization with six strata defined by the child’s age group (6 months–5 years, 6–11 years old, 12–17 years old; defined by the age of the child with the most recent birthday) and sex (boy, girls). Each eligible, consented participant was assigned to one of two study conditions: (1) digital storytelling or (2) information control group. Participants were invited to attend a group viewing session based on their group assignment. During this session, each participant was invited to complete a baseline questionnaire (T1), view the four videos (digital stories or nutrition information videos), followed by the COVID-19 factual video, and then immediately complete a post-intervention (T2) questionnaire. Participants also completed the narrative qualitative assessments (e.g., identification and transportation) after each video. The intervention and questionnaires were built and administered using Qualtrics (23), a secure online data collection and management platform. Two months later, we contacted all participants and asked them to complete a follow-up assessment (T3) to determine if parents had their child(ren) vaccinated against COVID-19. To compensate them for their time and effort, participants received a $20 USD incentive for each survey completed, with total compensation amounting to $60 across the study.

#### 2.2.4. Digital Storytelling Intervention

The digital storytelling video intervention consisted of the four most persuasive, culturally relevant, and personal stories selected by community advisory board members, and each story lasted approximately 3 min. See Table 1 for the digital story overviews. All stories were developed in Spanish, with English subtitles for broader accessibility.

#### 2.2.5. Control Group

Information control materials were designed to parallel digital storytelling intervention materials in terms of format (four videos) and duration of presentation (3 min for each video). The information control videos contained only information about childhood nutrition, drawn from American Heart Association [34,35] and United States Department of Agriculture [36] resources. All information control videos were developed in Spanish.

After the intervention, both groups watched a Spanish-narrated video about COVID-19 vaccine myths and facts, which was based on CDC fact sheets [37].

#### 2.2.6. Focus Group Discussions

We organized three focus groups, each consisting of 6–8 Hispanic parents, yielding a total of 17 participants from the intervention arm (see Appendix A). All focus groups were audio-recorded and transcribed by a professional transcription company. The transcripts were used to conduct qualitative analysis to explore participants’ perceptions of the effectiveness of digital storytelling intervention and its impact on vaccine decision-making for their children. We compensated all focus group participants $30.

### 2.3. Measures

Study materials had previously been translated into Spanish by a professional translator. The study materials were first developed in English and then translated and back-translated for review by community health workers. The T1 assessment included items measuring sociodemographic characteristics, Theory of Planned Behavior constructs (attitudes, subjective norms, and behavioral control) [38], and COVID-19 vaccine hesitancy and intentions [39]. The T2 assessment included the same measures that assessed the Theory of Planned Behavior constructs. The T2 assessment also included the Narrative Quality Assessment [40] to capture participants’ reactions to each story included in the intervention. Vaccine uptake was assessed at 2 months post-intervention (T3).

#### 2.3.1. Feasibility

Feasibility was assessed by comparing the observed participation rate (percentage of eligible individuals agreeing to participate), retention rate (proportion retained through follow-up), and involvement to a priori benchmarks (80% for each of the three indicators). We also examined between-group differences in rates of retention and missing data at T2 and T3.

#### 2.3.2. Acceptability

Acceptability of the intervention was examined through post-intervention focus group discussions with parents in the intervention arm. During these sessions, participants were invited to share their general impressions of taking part in the intervention, their reactions to viewing other parents’ digital stories, and which narratives they felt personally resonated with or seemed less applicable to their own experiences. Parents were also asked to reflect on the appropriateness of presenting stories about vaccination decisions and to provide their views on whether similar digital narratives might be useful or meaningful for other parents considering COVID-19 vaccination for their children.

#### 2.3.3. Sociodemographic Characteristics

Questions were used to assess parent’s age, sex, relationship to child, education, employment, income, language spoken at home, number of children, insurance status, and COVID-19 vaccine history.

#### 2.3.4. Acculturation

Acculturation was measured using a five-item scale. The items assessed language spoken, language read, place of residence, current circle of friends, and identification with a Latino/Hispanic background [41].

#### 2.3.5. Parent’s Attitudes About Vaccinating Their Child(ren) Against COVID-19

Parents’ perspectives on vaccinating their children against COVID-19 were evaluated using a four-item scale designed to capture vaccine-related attitudes [38] (Cronbach’s α = 0.72 at T1; 0.81 at T2) adapted to reflect attitudes toward COVID-19 vaccination for children (e.g., “I believe that getting the COVID-19 vaccine is beneficial for my child.”).

Items for all Theory of Planned Behavior construct measures (attitudes, perceived social norms, perceived behavioral control) were meant to use a 7-point Likert-type response scale ranging from 1 (*Strongly disagree*) to 7 (*Strongly agree*); however, due to programming errors, the response option of 6 (*Somewhat agree*) was omitted at T1, and the response option of 7 (*Strongly agree*) was omitted at T2. To align item scoring across time points, scores of 7 at T1 were recoded to 6. Each composite measure was computed as the mean of the available item scores.

#### 2.3.6. Parent’s Perceived Social Norms About Vaccinating Their Child(ren) Against COVID-19

To capture how parents viewed the expectations of important others about vaccinating their children, we used a five-item validated scale on vaccination norms [38], modified to specifically address COVID-19 immunization for children. An example item was: “My child’s doctor believes my child should receive the COVID-19 vaccine.” Reliability of the adapted measure was strong, with Cronbach’s alpha coefficients of 0.79 at baseline (T1) and 0.89 at follow-up (T2).

#### 2.3.7. Parent’s Perceived Behavioral Control to Vaccinate Their Child(ren) Against COVID-19

Parents’ sense of control over the decision to vaccinate their child(ren) against COVID-19 was measured with a four-item validated scale [38]. Items were updated to reflect COVID-19 vaccine decision-making for children. For instance: *“It is entirely my choice whether my child gets the COVID-19 vaccine.”* This instrument demonstrated high internal consistency (α = 0.82 at T1; α = 0.90 at T2).

#### 2.3.8. Parent’s COVID-19 Vaccine Hesitancy

Baseline vaccine hesitancy was measured using a modified version of the Parent Attitudes about Childhood Vaccines questionnaire, adapted to COVID-19 vaccines. The 15-item instrument captures three domains: (1) reported vaccination behaviors (2 items), (2) beliefs about vaccine safety and effectiveness (4 items), and (3) general vaccine attitudes (9 items). This tool has been widely applied to assess variations in parental confidence toward childhood immunizations. The scoring rubric for the Parent Attitudes About Childhood Vaccines is somewhat complex. Briefly, where applicable, raw items scores are rescaled such that “vaccine-hesitant” responses are coded as 2, expressions of uncertainty (e.g., *don’t know*, *not sure*) are either assigned a score of 1 (13 items) or coded as missing (2 items), and “non-hesitant” responses are coded as 0. The assigned codes are summed to produce a raw total, which is then rescaled to yield scores ranging from 0 to 100. Further details are available from the authors upon request [39].

#### 2.3.9. Parent’s Intentions to Vaccinate Child(ren) Against COVID-19

A single previously validated survey item developed based on Theory of Planned Behavior constructs and adapted to reflect COVID-19 vaccination intentions (“I intend to vaccinate my child against COVID-19 in the next two months.”) was used to assess parents’ intention to vaccinate their children at all 3 time points [38].

#### 2.3.10. Child’s COVID-19 Vaccine Uptake

Child’s COVID-19 vaccine uptake was measured using a single item asking whether the child received a COVID-19 vaccine dose since T2, with response options of yes and no.

#### 2.3.11. Vaccine Barriers

Vaccine barriers were assessed using an 8-item rank order measure listing factors preventing participants from vaccinating their child against COVID-19 (e.g., “I find it inconvenient to have my child vaccinated against COVID-19”). The scale was adapted from a previous study to reflect COVID-19 vaccines [38].

#### 2.3.12. Reactions to Digital Story and Content

The Narrative Quality Assessment scale, used to assess parents’ perceptions of the digital stories in the intervention group only (*n* = 40), comprises two 6-item subscales: identification with story and engagement/transportation. Item response options range from 1 (Disagree a lot) to 5 (Agree a lot); a higher score in subscales indicates stronger identification (with characters, story, and cultural elements), and a higher level of transportation (emotional engagement). Psychometric properties of the scale were reported in our previous work [40]. In the current sample, the subscales showed good internal consistency reliability, with mean Cronbach’s alpha values of 0.87 for identification and 0.96 for engagement/transportation, respectively, across the 4 videos. Generalizability coefficients derived from variance components analyses of item scores also indicated good reliability for both subscales (0.91 for identification and 0.95 for engagement/transportation).

### 2.4. Statistical Analysis

Quantitative data were managed and analyzed using R version 4.4.2 [42]. We conducted univariate (e.g., means, frequencies) and correlation analyses to describe distributions of, and association among, key variables. To explore changes in Theory of Planned Behavior constructs and vaccine intentions from pre- to post-intervention, we estimated intervention effect sizes with multivariable linear regression models, using an ANCOVA-type approach, where the outcome at T2 (or T3) is predicted from group assignment (Digital Storytelling vs. Information Control), adjusting for the T1 value on the outcome measure. To explore the between-arm differences in post-T2 vaccination rates, we estimated the odds ratio and risk ratio for the association between study arm and parent-reported vaccination behavior.

### 2.5. Qualitative Analysis

All focus group sessions with intervention participants were digitally recorded, translated, and transcribed into English prior to analysis. The team employed an inductive, team-based thematic analysis to guide the qualitative process [43,44]. Initially, each member of the research team (SWK, AK, FL) read through all transcripts, with the senior author leading the development and refinement of the codebook. This process involved adding new codes, collapsing redundant ones, and iteratively revising definitions. A lead investigator also provided structured training in qualitative coding to a doctoral student researcher. Together, they independently coded transcripts on a weekly basis, then met to reconcile differences and refine coding decisions until consensus was reached. Following this step, the team synthesized codes, integrated the ATLAS.ti output, and generated thematic summaries across transcripts. The final stage involved consolidating findings into a comprehensive narrative that captured key themes and patterns across participant accounts.

## 3. Results

### 3.1. Sample Characteristics

#### 3.1.1. Digital Storytelling Workshop (Phase I)

In the community sample of 10 workshop participants (mean age 47.6 years; *SD = 9.8* years), 100% identified as Hispanic/Latino, and Spanish was their primary language spoken at home. The participant sample also consisted of 20% of participants who reported being from a suburb near a large city, and 80% reported being from a large city.

#### 3.1.2. Intervention (Phase II)

As summarized in Table 2, most participants in the community sample of 80 parents were mothers (81%), identified as female (91%), identified as Hispanic or Latino (91%), reported living in a major city (85%), reported speaking only Spanish at home (81%), and reported being either married or living with a partner (75% total). Nearly three-fifths of participants reported annual household incomes of $50,000 or less. Of the children living with the participants (median = 2), 66% were girls. Half of the children had been vaccinated against COVID-19, and large majorities had a primary care provider (89%) and health insurance coverage (81%).

### 3.2. Feasibility

**Figure 1:**
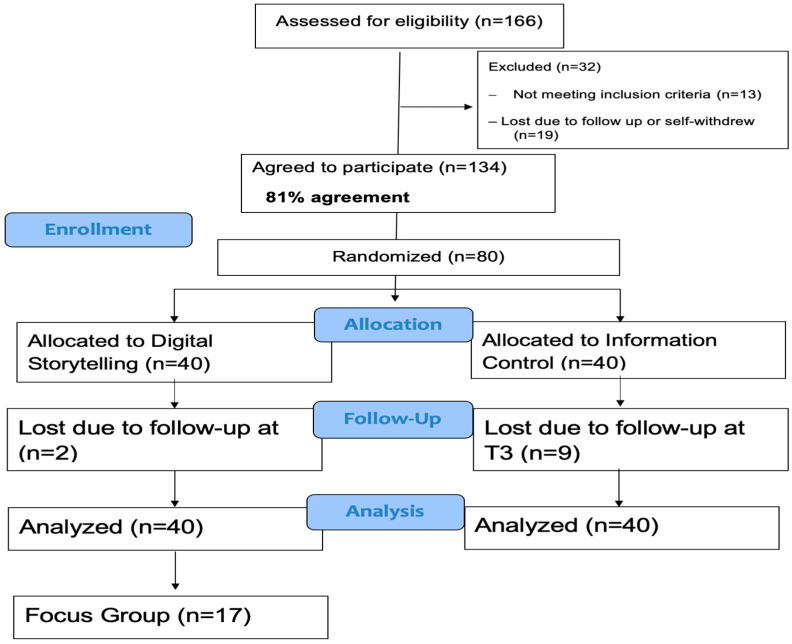
Consort Diagram.

Figure 1 illustrates the study flow and addresses the feasibility of recruitment and retention benchmarks. Among parents approached and reached (*n* = 166), 19 (11.4%) declined to take part because of loss of communication upon follow-up or self-withdrew their name, and 13 (7.8%) were ineligible due to not meeting eligibility criteria (e.g., child already received all COVID-19 vaccines or over the age of 18). Of the remaining 134 participants (80.8%), 80 agreed to participate in the feasibility trial. Half (*n* = 40) were randomized to the intervention group, and half (*n* = 40) were randomized into the control group. Of those enrolled in the feasibility trial, 69 (86.2%) participated at T3 and completed the T3 assessments. Reasons for not completing T3 were due to loss of communication upon follow-up. For our feasibility benchmarks, all recruitment, retention, and data completion rates exceeded our goal of 80% recruitment and 80% retention.

### 3.3. Acceptability

#### 3.3.1. Exploration of Between-Arm Differences in Patterns of Change and Vaccination Rates

As shown in Table 3, except for intention to vaccinate one’s child, patterns of change in measures of Theory of Planned Behavior constructs from T1 to T2 did not differ markedly across study arms. For the construct of intention, however, the improvement in mean scores in the intervention group (D = +1.3 at T2; 1.2 at T3) was at least twice that seen in the information control group (D = +0.6 at T2 and T3). Correlations between T1 (baseline) and T2 assessments were moderate to strong for attitudes, perceived social norms, and perceived behavioral control (*rs* = 0.44–0.70) and small to moderate for intentions at T2 (*r* = 0.39) and T3 (*r* = 0.26). Results of the linear regression analyses (see Table 4) show that the magnitude of the intervention effects on Theory of Planned Behavior constructs were generally small (|*d*|*s* ≤ 0.30), with the effects on intention at T2 being moderate (*d* = 0.41). At T3, parents in the intervention group were much more likely to report having their children vaccinated (*n* = 18 out of 36) than were parents in the control group (*n* = 5 out of 31); *OR* = 5.20, 95% CI = 1.63–16.57; *RR* = 3.10, 95% CI = 1.30–7.37.

#### 3.3.2. Exploration of Associations of Narrative Quality Assessment Measures with Change in TPB Constructs

In the intervention group (*n* = 40), examination of partial correlations between narrative quality assessment measures at T2 and change in Theory of Planned Behavior measures from T1 to T2 were generally weakly to moderately positive (*rs* = 0.24–0.55; see Table 5), except for perceived behavioral control (*r* = 0.06 and 0.00, for identification and engagement/transportation, respectively). Associations were stronger for identification than for engagement/transportation. Change in intention to vaccinate from T1 to T3 was weakly associated with both narrative quality assessment measures (*rs* = 0.27 and 0.24, for identification and engagement/transportation, respectively).

### 3.4. Qualitative Results

Participant responses from the focus group sessions highlighted the overall acceptability of the digital storytelling intervention. Several participants described being emotionally affected by the stories, underscoring the power of narrative in communicating health-related information. One participant remarked,

*“The truth is that those videos move anyone and make you reflect.”*

(Focus Group [FG] 2, Participant [P] 1).

Another emphasized why the stories were impactful. Hearing personal accounts from their community made the information more credible and also emotionally resonated with some participants:

*“Yes, when someone tells their story, it’s more believable… We are seeing the expression of anguish when the problem happens to you, the virus, the disease, and the joy when we are victorious. Or when there was a death, then you become more conscious and you say, ‘I don’t want to see myself suffer the way this person is suffering. Yes, I am going to get [the vaccine] because, as a result of [getting] the vaccine, the lady is alive.”*

(FG 3, P1)

Another participant further described feeling connected to the videos due to identifying with the storytellers,

*“I was affected by the stories because I lived through many of those things… These are moments when we thank God for the beauty of having been together. Maybe it had to be that way because he was a very dear uncle to us. That’s what moved me about the stories.”*

(FG 1, P 3)

Participants generally agreed that sharing personal narratives as digital stories can be a powerful way to ensure the dissemination of relatable, trustworthy, and impactful vaccine information. One participant appreciated that digital storytellers came from her community.

*“For me, they [the digital stories] were also good because they give us a focus on what is happening in the community and what each family is experiencing. The negative things that we hear are just speculation. But this is something more concrete … [it’s an] experience of a person or a family that has already lived through it. I think it’s positive to see how these people came out of this disease or pandemic.”*

(FG 1, P2)

In contrast, one participant described never changing perspectives about the vaccine.

*“I’m not convinced by this. If I don’t know that person personally, I don’t know if they were paid to say that. I’m not going to put something in my body that I don’t know personally. I don’t like going to the doctor, and at the same time, if I’m going to get sick or die, you never know. No. That’s it.”*

(FG 3, P 5)

Overall, most focus group participants expressed strong support for DSTs as a compelling and trustworthy approach to vaccine education, particularly when the stories reflected lived experiences within their communities. Many felt that DSTs had the potential to extend beyond COVID-19 messaging to other public health issues.

## 4. Discussion

In this study, we sought to address a significant public health problem, COVID-19 vaccine hesitancy. This study is, to the best of our knowledge, the first to examine the feasibility, acceptability, and explore patterns of change in Theory of Planned Behavior constructs, vaccine hesitancy, and vaccination behavior in an intervention group relative to those in a control group, among Hispanic parents of children who are not up to date with CDC-recommended COVID-19 vaccine doses. Our findings demonstrate that the intervention was both feasible and well-received, largely facilitated through a community-based approach involving partnerships with trusted community health workers. High recruitment and retention rates highlight its practical implementation potential. Furthermore, the intervention was deemed acceptable, as reflected in positive focus group feedback, where participants expressed appreciation for engaging with digital stories of other parents, identifying stories they related to, and finding value in the emotional and relatable content. Qualitative data further supports these findings, emphasizing the relatability, trustworthiness, and emotional impact of the digital stories. Participants reported that they identified personally with the storytellers and favored the narrative style over purely factual information. This was consistent with previous research [23,24,25,45], showing the effectiveness of culturally relevant digital storytelling interventions in vaccine promotion. For example, a recent study by Dunlap et al. [25] demonstrated that combining storytelling with social media reduced COVID-19 vaccine hesitancy. These results align with our findings that culturally resonant narratives are powerful tools for building trust and shaping vaccine perception.

Although effect sizes on Theory of Planned Behavior constructs were generally small to moderate, the intervention had a more notable impact on vaccination intentions at T2 and T3, with parents in the intervention group showing larger increases than controls. At T3, relative to control group parents, intervention group parents were over 3 times as likely to have had their children vaccinated, indicating potential for behavioral change. Both groups had received basic factual COVID-19 vaccination information; these results suggest that the addition of digital storytelling, rooted in trust and cultural relevance, can positively influence perceptions and behaviors regarding child vaccination, key factors within this community [23,25,46,47]. Our analysis of narrative quality, specifically identification and transportation, revealed positive partial correlations with changes in Theory of Planned Behavior measures from T1 to T2. We co-created these stories with Hispanic parents of vaccinated children, ensuring they are linguistically and culturally resonant, with characters that reflect the characteristics (appearance, voices, language, family photos) of other Hispanic parents. This similarity fosters identification and transportation, which can lead to changes in attitudes, beliefs, and vaccination intent among Hispanic parents whose children are not yet vaccinated. Viewing stories of Hispanic parents sharing personal experiences about overcoming vaccine hesitancy may effectively address common concerns and counterarguments, resonating with other hesitant parents or guardians and positively influencing their beliefs and confidence regarding vaccination. Although this study does not elucidate the specific mechanisms by which digital stories influence intentions and behaviors, future research with larger samples can explore these pathways theoretically and empirically.

Participants reported increased vaccination intentions at T2, and their subsequent behaviors by T3 underscore the promising potential DST as an intervention tool for improving vaccine uptake. The effectiveness of digital storytelling in empowering young Black adults to make decisions about the COVID-19 vaccine [23] and the results of the current study point to the promise of digital storytelling in non-White populations. This highlights the importance of culturally based health communication strategies surrounding vaccines and is consistent with a scoping review that found digital storytelling interventions help improve knowledge and attitudes regarding complex health issues of young adults of color [23]. Due to the dissemination of misinformation about vaccines, hearing stories from their own community members can help marginalized communities like the Hispanic population build trust in vaccines and combat vaccine hesitancy. Future research should investigate the mechanisms through which digital storytelling influence vaccine decision-making among Hispanic parents, focusing on emotional engagement, identification with storytellers, and changes in Theory of Planned Behavior constructs. Larger-scale trials are necessary to evaluate the effectiveness of digital storytelling interventions in real-world settings and assess their long-term impact on vaccination rates.

Overall, our study demonstrates the potential of digital storytelling as a culturally tailored, community-supported approach to reducing parental vaccine hesitancy and increasing COVID-19 vaccine uptake among Hispanic families. Future research should focus on whether these findings can be replicated in larger, more diverse samples to better understand the mechanisms through which digital stories influence attitudes and behaviors. Investigating variations in storytelling formats—such as the use of entertainment education like telenovelas or interactive digital stories—may help identify the most effective strategies for engagement and behavior change. Clinically, these findings underscore the importance of leveraging trusted community members and culturally resonant narratives to address vaccine hesitancy. Healthcare providers and public health practitioners should consider integrating digital storytelling into community outreach, especially in low-literacy and limited-English-proficiency populations. Training community health workers to facilitate and disseminate these stories could further enhance trust and message receptivity, ultimately fostering informed decision-making and improving vaccination rates. Further studies should explore the applicability of this approach within diverse cultural and faith-based communities to determine whether similar successes can be replicated. These efforts are critical to developing more robust, culturally tailored strategies that promote impactful vaccine decisions for youth and future generations.

### Limitations

Study findings may be limited to low-income, limited-English-proficient Hispanic families as opposed to the broader population of vaccine-hesitant Hispanic families. Participants’ self-identification varied (e.g., “Latino,” country of origin, or White ancestry), which may limit consistency and comparability across studies. Delivering the intervention in small groups may introduce group dynamics such as groupthink and peer influence if participants discussed the study among themselves. However, we believe delivering the intervention in-person, in a group setting, with the help of bilingual research team members, as opposed to completing the intervention alone and at home, may have helped reduce issues related to the digital divide and potential limited health literacy. Additionally, individuals who receive health education from this community health worker organization in small group settings and in-person settings. The scalability of this intervention may be more challenging since it was not conducted fully online. Conducting this study as a larger effectiveness trial would require training more community health workers to expand the study beyond initial settings. While vaccination intention improved more in the intervention group than the control, we did not stratify participants by baseline hesitancy, making it difficult to determine how opinions shifted. Future studies should use larger samples to track changes among initially hesitant parents. Furthermore, we used the Parent Attitudes about Childhood Vaccines scale, which is specific to childhood immunization decisions. This limits comparability with studies that have used broader instruments such as the Vaccination Attitudes Examination scale. Future research should consider including multiple scales to determine whether different tools capture distinct dimensions of vaccine attitudes and responsiveness to intervention. Despite these limitations, our findings contribute to the growing evidence supporting narrative-based interventions as effective tools for promoting positive health behaviors.

## 5. Conclusions

This pilot demonstrates the promise of community-driven digital storytelling interventions in addressing vaccine hesitancy among Hispanic parents. Building on these preliminary findings, future work could examine the use of digital storytelling by formerly vaccine-hesitant parents to reduce hesitancy for other vaccines such as influenza and HPV. Larger-scale trials could evaluate the scalability and sustained impact of digital storytelling in diverse populations and settings. Ultimately, this innovative approach may serve as a scalable, low-cost strategy for improving childhood and adolescent immunizations and for future rapid vaccination efforts, offering a culturally relevant tool to combat parental vaccine hesitancy across various contexts and outbreaks.

## Figures and Tables

**Table 1:** Description of Digital Stories.

Digital Storyteller	Story Content
Digital Storyteller 1	Story of a mother whose child nearly died because she wasn’t vaccinated against COVID-19.
Digital Storyteller 2	Story of a mother who was hospitalized because she became ill from COVID-19.
Digital Storyteller 3	Story of a pregnant mother who experienced loss during her pregnancy after losing her own mother of COVID-19
Digital Storyteller 4	Story of a woman who changed her perspective towards COVID-19 vaccination after her younger daughter asked to be vaccinated.

**Table 2:** Characteristics of intervention (Phase II) sample (*n* = 80).

Characteristic	Information Control (*n* = 40)	Intervention (*n* = 40)	Overall (*n* = 80)
Participant relationship to child ^a^			
Mother	30 (75.0%)	35 (87.5%)	65 (81.3%)
Father	1 (2.5%)	1 (2.5%)	2 (2.5%)
Other	9 (22.5%)	4 (10.0%)	13 (16.3%)
Participant age (yrs) ^b^	44.0 (13.5)	45.0 (10.3)	44.5 (11.9)
Participant sex ^a^			
Male	1 (2.5%)	1 (2.5%)	2 (2.5%)
Female	39 (97.5%)	39 (97.5%)	78 (97.5%)
Participant race/ethnicity ^a^			
White	2 (5.0%)	3 (7.5%)	5 (6.3%)
Hispanic/Latino	37 (92.5%)	36 (90.0%)	73 (91.3%)
Other	1 (2.5%)	1 (2.5%)	2 (2.5%)
Language(s) spoken at home ^a^			
Spanish only	33 (82.5%)	32 (80.0%)	65 (81.3%)
English and Spanish	7 (17.5%)	8 (20.0%)	15 (18.8%)
Type of community (self-reported) ^a^			
Major City	34 (85.0%)	34 (85.0%)	68 (85.0%)
Rural Area	2 (5.0%)	0 (0.0%)	2 (2.5%)
Suburban, near major city	4 (10.0%)	6 (15.0%)	10 (12.5%)
Participant marital status ^a^			
Married	22 (55.0%)	30 (75.0%)	52 (65.0%)
Single	6 (15.0%)	4 (10.0%)	10 (12.5%)
Live with a partner	5 (12.5%)	3 (7.5%)	8 (10.0%)
Widowed	3 (7.5%)	2 (5.0%)	5 (6.3%)
Separated	4 (10.0%)	1 (2.5%)	5 (6.3%)
Children under 18 years old living with participant ^c^	2.00 (1.00, 3.00)	2.00 (1.00, 3.00)	2.00 (1.00, 3.00)
Missing	0 (0.0%)	1 (2.5%)	1 (1.3%)
Participant education ^a^			
8th grade or less	9 (22.5%)	9 (22.5%)	18 (22.5%)
Some high school	5 (12.5%)	4 (10.0%)	9 (11.3%)
High school graduate or GED	15 (37.5%)	15 (37.5%)	30 (37.5%)
Some college or 2-year degree	7 (17.5%)	9 (22.5%)	16 (20.0%)
4-year college degree	2 (5.0%)	1 (2.5%)	3 (3.8%)
More than 4 years of college or advanced degree	2 (5.0%)	2 (5.0%)	4 (5.0%)
Participant employment status ^a^			
Full time	10 (25.0%)	8 (20.0%)	18 (22.5%)
Part time	4 (10.0%)	13 (32.5%)	17 (21.3%)
Not Employed	22 (55.0%)	19 (47.5%)	41 (51.3%)
Retired	4 (10.0%)	0 (0.0%)	4 (5.0%)
Household income level ^a^			
≤$30,000	10 (25.0%)	13 (32.5%)	23 (28.8%)
$30,001–50,000	13 (32.5%)	9 (22.5%)	22 (27.5%)
$50,001–75,000	3 (7.5%)	5 (12.5%)	8 (10.0%)
$75,000–100,000	1 (2.5%)	0 (0.0%)	1 (1.3%)
Refused	13 (32.5%)	13 (32.5%)	26 (32.5%)
Child age (yrs) ^b^	8.5 (4.1)	9.8 (5.0)	9.2 (4.6)
Child sex ^a^			
Female	26 (65.0%)	27 (67.5%)	53 (66.3%)
Male	14 (35.0%)	13 (32.5%)	27 (33.8%)
Child’s lifetime COVID-19 diagnosis ^a^			
Yes	13 (32.5%)	20 (50.0%)	33 (41.3%)
Had symptoms, but not formally diagnosed	5 (12.5%)	5 (12.5%)	10 (12.5%)
No	22 (55.0%)	13 (32.5%)	35 (43.8%)
Don’t know	0 (0.0%)	2 (5.0%)	2 (2.5%)
Child’s lifetime COVID-19 vaccination status ^a^			
Yes	17 (42.5%)	22 (55.0%)	39 (48.8%)
No	22 (55.0%)	13 (32.5%)	35 (43.8%)
Don’t know	1 (2.5%)	5 (12.5%)	6 (7.5%)
COVID-19 vaccine doses child has received ^a^			
Original doses (2 for Pfizer and Moderna), but no booster doses	17 (42.5%)	22 (45%)	39 (48.8%)
None, but I intend to vaccinate him/her.	7 (17.5%)	3 (7.5%)	10 (12.5%)
None, and I’m not going to vaccinate him/her.	13 (32.5%)	12 (30.0%)	25 (31.3%)
Don’t know	3 (7.5%)	3 (7.5%)	6 (7.5%)
Where child is likely to get COVID-19 vaccine ^a^			
Community health fair	10 (25.0%)	8 (20.0%)	18 (22.5%)
Doctor’s office	21 (52.5%)	22 (55.0%)	43 (53.8%)
Community pharmacy	6 (15.0%)	4 (10.0%)	10 (12.5%)
Other	3 (7.5%)	6 (15.0%)	9 (11.3%)
Child has a primary care provider ^a^	35 (87.5%)	36 (90.0%)	71 (88.8%)
Child has health insurance coverage ^a^	35 (87.5%)	30 (75.0%)	65 (81.3%)

^a^ *n* (%). ^b^ mean (standard deviation). ^c^ median (1st quartile, 3rd quartile).

**Table 3:** Observed means, standard deviations, and pre-post correlations for study outcome measures by study arm and time point.

	T1			T2			T3			T1–T2	T1–T3
Study Arm	*n*	M ^b^	(*SD* ^c^)	*n*	M	(*SD*)	*n*	M	(*SD*)	*r*	*r*
TPB ^a^: Positive vaccine attitudes										0.44	-
Information control	40	3.7	(1.1)	40	4.2	(1.3)	-	-	-		
Intervention	39	3.9	(0.8)	40	4.3	(1.4)	-	-	-		
TPB: Perceived positive social norms										0.69	-
Information control	40	3.9	(1.4)	39	4.2	(1.3)	-	-	-		
Intervention	39	4.1	(1.2)	40	4.4	(1.4)	-	-	-		
TPB: Perceived behavioral control										0.70	-
Information control	40	4.4	(1.3)	39	4.9	(1.2)	-	-	-		
Intervention	40	4.4	(1.4)	40	4.6	(1.5)	-	-	-		
TPB: Intention to have child vaccinated										0.39	0.26
Information control	40	3.3	(2.1)	40	3.9	(2.4)	30	3.9	(2.3)		
Intervention	40	3.4	(2.2)	39	4.7	(2.1)	37	4.6	(2.4)		

^a^ Theory of Planned Behavior. ^b^ mean. ^c^ standard deviation.

**Table 4:** Model-estimated post-intervention means, 95% confidence intervals, and intervention effect size (Cohen’s *d*) estimates for Theory of Planned Behavior (TPB) measures.

Study Arm	M	95% CI	*d*
T2 TPB: Positive vaccine attitudes			
Information control	4.2	(3.8, 4.6)	0.14
Intervention	4.4	(4.0, 4.7)	
T2 TPB: Perceived positive social norms			
Information control	4.3	(4.0, 4.7)	0.06
Intervention	4.4	(4.1, 4.7)	
T2 TPB: Perceived behavioral control			
Information control	4.9	(4.5, 5.2)	−0.27
Intervention	4.6	(4.3, 4.9)	
T2 TPB: Intention to have child vaccinated			
Information control	3.9	(3.3, 4.5)	0.41
Intervention	4.7	(4.1, 5.4)	
T3 TPB: Intention to have child vaccinated			
Information control	3.9	(3.1, 4.8)	0.30
Intervention	4.6	(3.9, 5.4)	

**Table 5:** Associations of Narrative Quality Assessment measures with change in the Theory of Planned Behavior (TPB) constructs from T1 to T2 and T3.

	Identification		Transportation	
TPB Construct	*r*	95% CI	*r*	95% CI
Positive vaccination attitudes	0.53	(0.26, 0.70)	0.35	(0.03, 0.58)
Perceived positive social norms	0.33	(0.01, 0.56)	0.24	(−0.08, 0.50)
Perceived behavioral control	0.06	(−0.26, 0.35)	0.00	(−0.30, 0.31)
Intention (T2)	0.55	(0.29, 0.71)	0.44	(0.14, 0.64)
Intention (T3)	0.27	(−0.07, 0.53)	0.25	(−0.08, 0.51)

## Data Availability

The raw data supporting the conclusions of this article will be made available by the authors on request.

## Appendix A

### Post-Intervention Focus Group Guide

Introduction:

Thank you for taking the time to participate in this study. Your responses will be used to explore the results of our research intervention aimed at increasing the willingness of Hispanic parents to vaccinate their children against COVID-19. The goal of this focus group is to gain a deep understanding of your overall experiences watching digital stories about COVID-19. We want to hear your feedback related to the stories you saw.

1. From the time you enrolled in the study, did your child receive more doses of the COVID-19 vaccine? Why or why not?
2. Did any of the stories catch your attention and resonate with you? If so, which one or which ones, and why?
  - One was from a mother whose son almost died because he was not vaccinated. (Fabiola’s story)
  - Another describes her change of perspective when her daughter asked for the vaccine (Juana’s story)
  - One was from a mother who, herself, was hospitalized because she was sick with COVID-19 (Julia’s Story)
  - One of them was a pregnant mother whose mother died of COVID-19, and experienced many losses during her pregnancy (Martha’s Story)
3. After watching the stories, what, if anything, changed in your views on the COVID-19 vaccine?
  - If so, why? In what way?
  - Was your decision to have your child get another dose of the COVID-19 vaccine influenced in any way by seeing the stories?
4. Before you saw other parents’ stories about COVID-19 vaccines, how open-minded were you about vaccinating your child?
  - What concerns, if any, did you have about giving your child COVID-19 vaccines before you saw the stories?
5. How might seeing similar stories about other types of vaccines help you decide whether to get that vaccine for your child?
6. In your opinion, how effective is it to show stories of parents who changed their perspective from hesitant to a confident perspective regarding vaccines? Why or why not?
7. What do you think about using personal stories, presented in short videos like what you saw in the study, to provide vaccine education (e.g., flu, MMR) to your children?
  - What would be the best way to tell these stories?
8. If this intervention didn’t convince you that vaccinating your child is a good option, what would you need to know? Whose perspective would you need to hear, or see (e.g., from a health care provider or a medical consultant)? Is there any other information you would have found helpful in wanting to vaccinate your child?
9. What else would you like to share at this time about your experience seeing these stories?

This concludes our interview. Thank you for your time and shared experience.
